# The Impact of Interdisciplinary Education on Skills and Attitudes of Surgery and Emergency Medicine Residents

**DOI:** 10.1055/s-0039-1681063

**Published:** 2019-03-20

**Authors:** Anastasia Kunac, Neil A. King, Ilya Ostrovsky, David Rytzarev, Aziz M. Merchant, Tiffany Murano

**Affiliations:** 1 Department of Surgery, Rutgers New Jersey Medical School, Newark, New Jersey

**Keywords:** interprofessional education, attitudes, skills, emergency medicine, general surgery, residents

## Abstract

**Background** Interdisciplinary education (IDE) has been proposed as a means to improve patient safety by enhancing the performance of diverse health care teams. The improved camaraderie between members of different specialties may enhance communication and can foster a more supportive and positive work environment.

**Objective** This study was aimed to assess the effect of IDE on the procedural skills of general surgery (GS) and emergency medicine (EM), as well as the perceptions that GS and EM residents have of one another.

**Methods** EM and GS residents participated in two separate IDE sessions (4 months apart) designed to teach extended focused assessment with sonography in trauma (e-FAST), tube thoracostomy, and complex wound closure. Surveys were administered to determine the effects that IDE had on confidence in performing bedside procedures, perceptions of IDE, and perceptions of one another's specialty. Survey responses were recorded using a 5-point Likert's scale.

**Results** Nine GS residents and 10 EM residents participated in the entire study. Significant improvements in the confidence levels of performing bedside procedures were noted among both groups of residents. We also report a significant improvement in the perceived respect and communication between EM and GS residents.

**Conclusions** Although further studies with a larger sample size are required, we have shown that IDE can improve the confidence levels of EM and GS residents in performing tube thoracostomy, e-FAST, and complex wound closure. These IDE sessions also improve the perceptions that the residents have of one another. IDE is a useful tool and may translate into improved consultation, collaboration, and patient care.

Modern health care warrants a team approach with members from multiple disciplines and different specialties within the medical profession. The cornerstone of any successful team approach is mutual respect and trust among its members. This invites collaboration, communication, and shared decision making. Studies have shown that poor communication and teamwork deficiencies are an important factor in poor clinical outcomes. 1 Teamwork in the health care context has been defined as “a dynamic process involving two or more health professionals with complementary backgrounds and skills, sharing common health goals and exercising concerted physical and mental effort in assessing, planning, or evaluating patient care. This is accomplished through interdependent collaboration, open communication, and shared decision making.” 2

Approximately 40% of all emergency departments (ED) involve a consultation of some kind. 3 4 This means that emergency medicine (EM) physicians must establish a relationship with the other services in the institution that relies on communication and trust. More specifically, in teaching institutions, EM residents frequently call upon the general surgery (GS) residents to consult on patients who present to the ED requiring surgical input or intervention. However, the high-stress environment of the ED combined with the demands of the residents' workload that may lead to poor interpersonal communication, contention between the EM and GS residents, and ultimately poor working relationships between the two specialties. Other factors that impact the interpersonal relationships between EM and GS residents with respect to ED consults and referrals are trust and familiarity. 5 There are also instances where EM and GS residents must work side-by-side in the ED, such as in the evaluation of the traumatically injured patients. This often includes the performance of extended focused assessment with sonography in trauma (e-FAST), tube thoracostomy, and management of its drainage system (Pleur-e-vac, Teleflex, Morrisville, NC), and complicated wound repair and management. The acquisition of these skills is a requirement for both EM and GS residents.

Traditionally certain procedures and skills are taught only to members of a certain discipline even though there may be a significant overlap with different specialists performing the same procedures. These skills are often taught according to a silo model, whereby more experienced members pass along skills to trainees within their own field. For surgery in particular, the foundations of surgical resident education have historically been a Halstedian's apprenticeship model which suggests that junior surgeons should learn from senior surgeons. 6 Other specialties utilize a similar teaching model where postgraduate trainees learn exclusively from faculty within their specialty. Interdisciplinary small group formats allows for the integration and transference of knowledge and skills of learners from various backgrounds.

Our goal was to compare the perceptions that EM and GS residents had of one another before and after interdisciplinary education (IDE) sessions. We hypothesized that by creating a combined educational activity outside of the clinical setting utilizing faculty from both specialties, EM, and GS residents would have improved attitudes toward the other specialty. We also hypothesized that after the IDE sessions, EM and GS residents would recognize the utility of learning from faculty outside of their own specific specialty.

## Methods

### Participants

This was a prospective study conducted at a level 1 trauma center. Approval was obtained from the “blinded for peer review” institutional review board. EM and GS residents were enrolled. EM residents were from the postgraduate year (PGY)-1 through PGY-4 classes, while GS residents were from the PGY-2 and PGY-3 classes.

### Intervention

Two separate IDE sessions were held. In the first session, e-FAST and tube thoracostomy skills were taught. In a group setting, PGY-2 and PGY-3 GS residents and PGY-1 and PGY-2 EM residents were taught how to perform an e-FAST examination by EM faculty, while the PGY-3 and PGY-4 EM residents were taught tube thoracostomy placement and drainage system by GS faculty and GS PGY-5 residents. The GS residents were not included in the tube thoroctomy training as learners for two reasons. The authors who are surgical faculty felt that the GS residents have extensive experience with chest tube placement, as well as management due to the various rotations within the residency curriculum. However, the EM residents have a limited exposure to performing tube thoracostomy throughout their training and have a requirement to demonstrate competency in this procedure during their training. In addition, EM residents are required to perform ten tube thoracostomy tubes during their training.

During the second session, GS residents underwent advanced training in e-FAST exam and bedside ultrasound facilitated by senior EM residents and faculty. A GS faculty member with assistance from a PGY-5 GS resident instructed the EM residents on advanced suturing techniques and wound management ( Table 1 ). The two sessions took place approximately 4 months apart during the same academic year.

**Table 1:** Schematic of the two interdisciplinary education (IDE) sessions indicating subject, learners, and instructors

	IDE session one		IDE session two (4 mo later)	
	Thoracostomy tube	e-FAST	Wound MAnagement	e-FAST and advanced US
**Learners**	EM: PGY-3, 4	GS: PGY-2, 3 EM: PGY-1, 2	EM: PGY-1, 4	GS: PGY-2, 3
**Instructors**	Surgery faculty and GS PGY-5	EM faculty	Surgery faculty and GS PGY-5	EM faculty and EM PGY-4

Abbreviations: e-FAST, extended focused assessment with sonography in trauma; EM, emergency medicine; GS, general surgery; IDE, interdisciplinary education; PGY, postgraduate year; US, ultrasound.

*Note* : This Table reflects the rating of the general surgery residents' perceptions of various aspects of their ultrasound skills before and after an interdisciplinary education session taught by emergency medicine faculty and residents.

Residents used a Likert's scale (1–strongly disagree, 2–disagree, 3–neutral, 4–agree, 5–strongly agree) and the ratings reflected in this table are for agree and strongly agree.

Consistency amongst sessions taught was maintained, as each education session has a prescribed set of didactic material for content delivery and educational goals and objectives for each session. Guidelines for content to be delivered was derived from standard textbooks in EM and surgery. Standards for tube thoracostomy placement were derived from course material for the American College of Surgeons Advanced Trauma Life Support (ATLS) course which is a required curricular component for both surgery and EM residency trainees.

### Measurements and Survey

A survey was designed to capture two major endpoints: the confidence levels of trainees performing various procedures (skills acquisition) and the perceptions of the impact interdisciplinary educational sessions have on interdisciplinary communication, respect, and patient care (perceptions). The responses were recorded on a 5-point Likert's scale (1–strongly disagree, 2–disagree, 3–neutral, 4–agree, and 5–strongly agree; or 1–never, 2–rarely, 3–sometimes, 4often, and 5–always). We administered the survey before any sessions were performed to identify the baseline perceptions. The same survey was administered after the sessions to assess the impact that working closely together had on the learners.

### Analysis

We compared the mean Likert's score from the baseline administration of the survey and the results following the second IDE session. We defined an improvement of perceptions as a reflection of stronger agreement with statements related to confidence in skills postintervention. Improvement, as it relates to attitudes and perceptions, was defined as less frequent negative interactions and more frequent positive interactions postintervention. Mean Likert's scores were compared using a paired *t* -test, with *p* -values less than 0.05 being considered significant. The positive responses (agree and strongly agree) and higher frequency responses were reflected as percentages of the total responses.

## Results

Nine GS residents were present for both IDE sessions on e-FAST and advanced ultrasound. The responses of the perceptions of GS residents are included only for residents who were present for both ultrasound sessions. Responses were recorded from 10 PGY-3 and PGY-4 EM residents during the chest tube session. Twelve EM residents were present for the advanced suturing skills and wound management session. A total of 10 EM residents were present for both sessions. Two EM residents were unavailable for both sessions due to clinical responsibilities, we only report their perception data as it relates to their performance of the skills taught during the session attended.

### Interdisciplinary Educational Impact on Skills

The GS residents showed a statistically significant improvement in their confidence and comfort levels with many aspects of the e-FAST. Prior to the IDE session, 0% of GS residents agreed or strongly agreed with feeling comfortable performing an e-FAST ( Table 2 ). Following the sessions 55% ( *p* = 0.001) of GS residents reported comfort with this procedure. GS residents reported increased confidence in interpreting images (11 vs. 67%, *p* = 0.01) and diagnosing a pneumothorax (11 vs. 67%, *p* = 0.005). No GS residents reported feeling confident sending a patient to the operating room based on the findings of the e-FAST prior to intervention. Following the IDE session 55% felt comfortable making this decision (0 vs. 55%, *p* = 0.02).

**Table 2:** General surgery residents: category–skills

Statement	Presession % (mean score) *n* = 9	postsession % (mean score) *n* = 9	*p* -Value
I feel comfortable with the ultrasound machine including how to turn it on, change to a different mode and cycle between the various probes	55 (3.5)	88 (4.2)	0.08
I feel comfortable performing an e-FAST in the setting of trauma	0 (1.77)	55 (3.66)	0.001 a
I feel comfortable in interpreting images I obtain using the ultrasound in the setting of trauma	11 (2.3)	67 (3.7)	0.01 a
I understand how to diagnose a pneumothorax using the ultrasound	11 (2.0)	67 (3.6)	0.005 a
I am confident sending a patient to the OR based on the findings of the e-FAST	11 (2.3)	55 (3.6)	0.02 a

Abbreviations: e-FAST, extended focused assessment with sonography in trauma; OR, operating room.

a Denotes statistical significance.

*Note* : This Table reflects the rating of the emergency medicine residents' perceptions of various aspects of their wound care, complicated wound closure and thoracostomy tube skills before and after an interdisciplinary education session taught by general surgery.

Residents used a Likert's scale (1–strongly disagree, 2–disagree, 3–neutral, 4–agree, 5–strongly agree) and the ratings reflected in this table are for agree and strongly agree.

EM residents reported a similar increase in their comfort level in both thoracostomy tube management and complex suture techniques. For example, there was a significant improvement in their comfort troubleshooting a malfunctioning thoracostomy tube, and setting up a chest drainage system (29 vs. 71%, *p* = 0.03, and 29 vs. 71%, *p* = 0.003; Table 3 ). The EM residents reported an enhancement in comfort with most aspects of wound debridement and closure. Over 90% of all EM residents (42 vs. 92%, *p* = 0.0006) felt comfortable performing a wound debridement following the IDE session.

**Table 3:** Emergency medicine residents: category–skills

Statement	Presession % (mean score) *n* = 12	Postsession % (mean score) *n* = 12	*p* -Value
I am comfortable with placing vertical mattress sutures	67 (3.6)	83 (4.25)	0.01 a
I am comfortable performing wound debridement	42 (3.16)	92 (4.16)	0.0006 a
I am comfortable with the sutures closure of complicated defects/wounds	58 (3.58)	83 (4.25)	0.02 a
I am comfortable placing subcuticular sutures	50 (3.41)	75 (4.08)	0.07
I am comfortable with placing horizontal mattress sutures	67 (3.58)	92 (4.5)	0.002 a
I understand how the Pleur-e-vac works and how to set up the Pleur-e-vac	29 (3.0)	71 (4.0)	0.003 a
I am comfortable trouble shooting a malfunctioning chest tube	29 (2.9)	71 (3.9)	0.03 a

*Note* : This Table reflects the rating of the general surgery residents' perceptions with respect to interdisciplinary training impact on education and patient care, communication with emergency medicine (EM) residents, and respect for EM residents, before and after an interdisciplinary education session taught by EM faculty and residents.

Residents used one of the following Likert's scales: 1–strongly disagree, 2–disagree, 3–neutral, 4–agree, 5–strongly agree; or 1–never, 2–rarely, 3–sometimes, 4–often, 5–always. The ratings reflected in this table are for percent of agree/strongly agree or often/always and the mean score.

a Denotes statistical significance.

### Interdisciplinary Educational Impact on Perceptions

Prior to the IDE sessions, 44% of GS residents agreed or strongly agreed that they enjoyed working with EM residents. The scores submitted by the GS residents increased to 89% ( *p* = 0.04; Table 4 ) following the IDE sessions. EM residents had less frequent instances in which they felt insulted by a GS consultant after the IDE session (80 vs. 40%, *p* = 0.02; Table 5 ).

**Table 4:** General surgery residents: category–perceptions

Statement	Presession % (mean score) *n* = 9	Postsession % (mean score) *n* = 9	*p* -Value
Interdisciplinary training impact on education			
Having emergency medicine faculty giving lectures and hands on training would benefit patient care and my knowledge ^†^	88 (3.55)	100 (4.33)	0.06
Learning procedures/examination skills directly from emergency medicine physicians is more beneficial than learning from someone in my own field ^†^	11 (2.11)	56 (3.55)	0.002 a
**Communication**			
Training with emergency medicine residents enhances communication of clinical information ^†^	56 (3.44)	100 (4.22)	0.06
I teach emergency medicine residents how to perform procedures ^††^	22 (2.77)	56 (3.33)	0.24
I learn how to perform procedures from emergency medicine residents ^††^	0 (1.88)	33 (2.88)	0.08
**Respect**			
I enjoy working with emergency medicine residents ^†^	44 (3.44)	89 (4.11)	.04
I have been insulted when being consulted by an emergency medicine resident ^†^	11 (2.2)	33 (2.3)	0.87
I feel respected by emergency medicine residents ^††^	33 (3.33)	67 (3.88)	0.27
**Patient care**			
Interdisciplinary education sessions enhance quality of consultations ^†^	67 (3.66)	100 (4.55)	0.002 a

a Denotes statistical significance.

*Note* : This Table reflects the rating of the emergency medicine (EM) residents' perceptions with respect to interdisciplinary training impact on education and patient care, communication with general surgery (G)S residents, and respect for EM residents. before and after an interdisciplinary education session taught by GS faculty.

Residents used one of the following Likert's scales: ^†^ 1–strongly disagree, 2–disagree, 3–neutral, 4–agree, 5–strongly agree; or ^††^ 1–never, 2–rarely, 3–sometimes, 4–often, 5–always. The ratings reflected in this Table are for the percent of agree/strongly agree or often/always and the mean score.

**Table 5:** Emergency medicine residents: category–perceptions

Statement	Presession % (mean score) *n* = 10	Postsession % (mean score) *n* = 10	*p* -Value
Interdisciplinary training impact on education			
Having surgery faculty giving lectures and hands on training would benefit patient care and my knowledge ^†^	80 (4.4)	100 (4.7)	0.27
Learning procedures/examination skills directly from surgeons is more beneficial than learning from someone in my own field ^†^	40 (3.4)	80 (4.2)	0.03 a
**Communication**			
Training with general surgical residents enhances communication of clinical information ^†^	90 (4.1)	100 (4.2)	0.67
I teach general surgery residents how to perform procedures ^††^	20 (2.0)	30 (2.9)	0.01 a
I learn how to perform procedures from general surgery residents ^††^	20 (2.3)	30 (3.1)	0.01 a
**Respect**			
I enjoy working with general surgery residents ^†^	30 (3.3)	50 (3.7)	0.26
I have been insulted when consulting a general surgery resident ^†^	80 (3.9)	40 (3.0)	0.02 a
I feel respected by general surgery residents ^††^	10 (3.0)	40 (3.5)	0.05 a
**Patient care**			
Interdisciplinary education sessions enhance quality of consultations ^†^	100 (4.4)	100 (4.3)	0.67

a Denotes statistical significance.

*Note* : Residents used one of the following Likert's scales: †1–strongly disagree, 2–disagree, 3–neutral, 4–agree, 5–strongly agree; or ††1–never, 2–rarely, 3–sometimes, 4–often, 5–always. The ratings reflected in this Table are for the percent of agree/strongly agree or often/always and the mean score.

Both EM and GS residents' attitudes toward the idea of learning skills and procedures from providers outside of their respective fields were more positive after the IDE sessions (EM: 40 vs. 80%, *p* = 0.03 and GS: 11 vs. 56%, *p* = 0.002). EM residents also reported a significant increase in the likelihood of learning procedures from GS residents and teaching procedures to GS residents (20 vs. 30%, *p* = 0.01 and 20 vs. 30%, *p* = 0.01, respectively). GS residents also felt that the sessions helped to enhance the quality of consultations (67 vs. 100%, *p* = 0.002).

## Discussion

The American Association of Colleges of Nursing (AACN) defines interdisciplinary education as “an educational approach in which two or more disciplines collaborate in the learning process with the goal of fostering interprofessional interactions that enhance the practice of each discipline.” The AACN further goes on to assert that IDE is “based on mutual understanding and respect for the actual and potential contributions of the disciplines.” 7

Our data show that residents from both disciplines benefited from the IDE sessions in both their abilities to perform procedures and their attitudes toward the other specialties. GS residents felt significant improvement not only in their ability to perform e-FAST, but also to act on those results with confidence. In addition, our study showed that EM residents had a significant improvement in their comfort with performing tube thoracostomy, complicated wound closure, and wound debridement following the IDE session instructed by a GS faculty member. This demonstrates that having sessions in which this educational “cross pollination” occurs can be highly effective at improving the confidence and skill levels of trainees. Conflicts between specialists created by traditional power structures have been identified as a barrier to implementing interdisciplinary collaboration. 8 Incorporating IDE into postgraduate training may foster professionals to be more receptive to the concept of not only team practice but also learning from one another in the future.

The most striking findings of our study pertain to the perceptions that GS and EM physicians have of one another, and the impact that IDE sessions have on these perceptions. As previously discussed, education and didactics are often take place in relative isolation with both learners and educators belonging to the same specialty.

Interestingly prior to the intervention, both groups of residents had generally favorable attitudes with respect to feeling that lectures and hands on training from faculty in the other specialty would benefit patient care and their knowledge. However, both groups of residents felt less favorably that learning procedures and examination skills directly from faculty in the other specialty would be more beneficial than their own specialty. These attitudes were more favorable after the intervention. This may be reflective of the effect that traditional learning models have on the learners and perhaps may foster the perception that expertize in a particular skill set may only exist within one's own specialty. Our study challenges this norm by utilizing faculty and senior residents from a nonsurgical specialty to teach surgery residents and learners in EM are taught by surgery faculty and senior residents. Faculty from each specialty modeled interdisciplinary camaraderie, mutual respect, and appreciation for one another's skill set.

With respect to communication, both groups of residents felt that training with other specialty's residents enhanced communication of clinical information. In general, both groups were either or neutral or did not feel that they taught or learned procedures from the other specialty's residents. These results may be due to the fact that the IDE sessions were primarily led by faculty with only senior EM residents assisting the ultrasound sessions. These results may also reflect the fact that cross-specialty education on a resident level is frequently limited in the actual clinical setting. This is because these procedures are often performed emergently and are taught under direct supervision by either a senior resident or a faculty member of the same specialty.

After the IDE sessions, the EM residents felt significantly less insulted when consulting a GS resident and more frequently respected by the GS residents. The GS residents had a nonsignificant improvement in their perception of feeling insulted when being consulted by EM residents and feeling respected by EM residents after the IDE sessions. There was a significant improvement in GS residents' enjoyment when working with EM residents while the EM residents had a statistically nonsignificant increase in their enjoyment working with GS residents. The increase in the enjoyment while working with one another during these IDE sessions may have a significant impact on fostering a sense of familiarity and trust–two key determinants of resident relationships. 5 This may also be reflective in the residents' feelings of being insulted less frequently.

After the intervention both groups of residents were agreed or strongly agreed that IDE sessions enhanced the quality of consultations. We feel that this is an important step in the way we educate our learners and foster a sense of collaboration and teamwork in the clinical setting.

## Limitations

Our study is subject to several limitations. The survey we used is not a previously validated instrument and may be subject to an unknown degree of inaccuracy secondary to poor reliability or validity. Based on feedback with regards to e-FAST skills following the first session, we repeated the intervention using a slightly different format. The feedback from the participants suggested that a smaller group would enhance the educational experience. Therefore, the second session was taught in smaller groups.

Although we had a small number of participants, we were able to follow the same trainees throughout the entire duration of the study. This allowed us to track each individual and provide consistency for our data. Even though all of our sessions involved IDE with members of one specialty providing instruction to learners of another specialty. Some learners did not interact with members from another specialty that were at a similar level of training during the sessions. This interaction may have proven to be more beneficial for the learners.

Additionally, since our study was not blinded and was performed in a single institution, the results may be influenced by some degree of cultural bias.

## Conclusion

Our results suggest that using an IDE model to teach skills and procedures that are common to both EM and GS residents can positively impact several factors, including perceptions, attitudes, and communication of residents between the two specialties. Although there were limitations to the study, our results suggest that collaborative education sessions allow residents to recognize the utility of learning from faculty outside of their own specialty. These are all key elements in providing a collaborative and collegial environment in the clinical setting. Future studies with increased number of learners will be needed to validate these findings.
